# Multilocus sequence typing of clinical *Borreliella afzelii* strains: population structure and differential ability to disseminate in humans

**DOI:** 10.1186/s13071-018-2938-x

**Published:** 2018-06-28

**Authors:** Floriane Gallais, Sylvie J. De Martino, Erik A. Sauleau, Yves Hansmann, Dan Lipsker, Cédric Lenormand, Emilie Talagrand-Reboul, Pierre H. Boyer, Nathalie Boulanger, Benoît Jaulhac, Frédéric Schramm

**Affiliations:** 1 EA7290 Early Bacterial Virulence: Lyme borreliosis Group, FMTS, University of Strasbourg, Strasbourg, France; 2 French National Reference Center for Borrelia, University Hospital Strasbourg, Strasbourg, France; 3 Groupe d’Étude de la Borréliose de Lyme (GEBLY), Strasbourg, France; 4 Public Health, Methods in Clinical Research Team, University Hospital Strasbourg, Strasbourg, France; 5 Infectious Disease Department, University Hospital Strasbourg, Strasbourg, France; 6 Dermatology Department, University Hospital Strasbourg, Strasbourg, France

**Keywords:** *Borrelia*, *Borreliella*, *Borreliella afzelii*, Multilocus sequence typing, Lyme borreliosis

## Abstract

**Background:**

Lyme borreliosis in humans results in a range of clinical manifestations, thought to be partly due to differences in the pathogenicity of the infecting strain. This study compared European human clinical strains of *Borreliella afzelii* (previously named *Borrelia afzelii*) using multilocus sequence typing (MLST) to determine their spatial distribution across Europe and to establish whether there are associations between *B. afzelii* genotypes and specific clinical manifestations of Lyme borreliosis. For this purpose, typing was performed on 63 strains, and data on a further 245 strains were accessed from the literature.

**Results:**

All 308 strains were categorized into 149 sequence types (STs), 27 of which are described here for the first time. Phylogenetic and goeBURST analyses showed short evolutionary distances between strains. Although the main STs differed among the countries with the largest number of strains of interest (Germany, the Netherlands, France and Slovenia), the *B. afzelii* clinical strains were less genetically structured than those previously observed in the European tick population. Two STs were found significantly more frequently in strains associated with clinical manifestations involving erythema migrans, whereas another ST was found significantly more frequently in strains associated with disseminated manifestations, especially neuroborreliosis.

**Conclusions:**

The MLST profiles showed low genetic differentiation between *B. afzelii* strains isolated from patients with Lyme borreliosis in Europe. Also, clinical data analysis suggests the existence of lineages with differential dissemination properties in humans.

**Electronic supplementary material:**

The online version of this article (10.1186/s13071-018-2938-x) contains supplementary material, which is available to authorized users.

## Background

Lyme borreliosis is the most prevalent tick-borne disease in humans occurring in the temperate regions of the Northern Hemisphere [1]. This multisystem disease is caused by infection with spirochaetal bacteria of the *Borrelia burgdorferi* (*sensu lato*) complex, previously included in the genus *Borrelia*. This genus was recently proposed to be subdivided into two genera: the emended genus *Borrelia*, containing only the causative agents of relapsing fever, and the genus *Borreliella*, containing the causative agents of Lyme borreliosis [2, 3]. To date, the genus *Borreliella* has been subdivided into 22 named species [4–6]. These bacteria are transmitted to humans and other vertebrate hosts *via* the bite of infected *Ixodes* spp. ticks, mainly *Ixodes ricinus* in Europe [7]. Five *Borreliella* species are mainly pathogenic to humans: *B. afzelii*, *B. burgdorferi*, *B. garinii*, *B. bavariensis* and, less often reported, *B. spielmanii* [5, 8]. All five species are present in Europe, although *B. afzelii* and, to a lesser extent, *B. garinii* predominate, whereas *B. burgdorferi* is the main species responsible for Lyme borreliosis in North America [7, 9].

*Borreliella* infection in humans can cause a range of clinical features, and patients may present with a variety of symptoms. These symptoms can vary according to the stage of the disease and the level of bacterial dissemination through the blood and tissues [7]. In most cases, a characteristic localized skin lesion, known as erythema migrans (EM), appears in the early stages of the disease and presents at the initial site of inoculation. After the process of bacterial dissemination, the infection can evolve into early disseminated disease in the forms of Lyme neuroborreliosis (NB), Lyme arthritis, or more rarely, multiple EM, borrelial lymphocytoma, or Lyme carditis. Other later manifestations include acrodermatitis chronica atrophicans (ACA), chronic Lyme arthritis, and late neurological manifestations [10]. Infection with certain *Borreliella* spp. has been associated with specific disseminated clinical manifestations: *B. afzelii* is associated with cutaneous symptoms, especially ACA, which is seldom observed with any other species; *B. garinii* and *B. bavariensis* are more frequently associated with NB; *B. burgdorferi* is associated with arthritic symptoms [7, 11, 12].

Multilocus sequence typing (MLST) is a molecular typing method used to characterize strains in terms of their pathogenicity and can be applied to *Borreliella* isolates. This reliable and highly discriminating typing method is based on sequence analyses of several housekeeping genes, where each haplotype is attributed to a sequence type (ST). The MLST scheme currently in use for *Borreliella* spp. consists of a sequence analysis of eight housekeeping genes (*clpA*, *clpX*, *nifS*, *pepX*, *pyrG*, *recG*, *rplB* and *uvrA*) [13]. Hanincova et al. [14] used this particular method to characterize 146 strains of *B. burgdorferi* isolated from human samples in New York and Wisconsin and reported the evidence of clonal complexes (CCs) with different dissemination properties. These results are supported by other studies, which also suggest that genotypes in a given species may not harbor the same dissemination property in the human body [15–19]. In Europe, most MLST studies have focused on differences in pathogenicity at the species level or have compared European and American *B. burgdorferi* strains [20, 21]. Only one published study has investigated intra-species differential pathogenicity among European strains of *Borreliella* spp. based on MLST data [15]. Among the strains included in that study, 135 *B. afzelii* strains were reported to have been isolated from human samples, mostly originating from the Netherlands (*n* = 79).

The present study proposed an approach that could characterize a large population of clinical *B. afzelii* strains originating from several European countries using MLST. Based on the resulting MLST data, the genetic diversity and phylogeny of 308 *B. afzelii* strains was analyzed. The aims were to compare strains originating from different European countries and to establish whether associations exist between the MLST profiles of *B. afzelii* and different clinical manifestations. Identifying the spatial distribution of specific bacterial genotypes and determining their associations with clinical symptoms of Lyme borreliosis is both epidemiologically and clinically relevant in our understanding of Lyme borreliosis.

## Methods

### Clinical isolates

In total, 308 strains were included in this study. Initially, 63 *B. afzelii* clinical isolates, a single isolate per patient, were obtained in culture and characterized using MLST. Most of these strains were isolated from specimens collected from patients infected in France (*n* = 61). One specimen was obtained from a patient infected in Germany and another from a patient probably infected in Switzerland. To increase the statistical power and sample size and to retrieve samples representing as many European countries as possible, additional clinical *B. afzelii* strains, previously analyzed using MLST, were also included. Sequence data on 121 strains were downloaded from the online MLST database. MLST data on a further 124 strains published by Coipan et al. were also added to the dataset, after excluding possible duplicates [15, 22]. Table 1 and Table 2 report geographical and clinical information on the 308 strains, respectively. Detailed information on the geographical origin and the clinical symptom as well as the sequence data relating to each strain are reported in Additional file 1: Database 1.

**Table 1:** European countries of origin of the clinical *B. afzelii* strains included in this study

Country of origin	Strains typed in this study	Strains typed by Coipan et al. [15]	Strains extracted from the MLST database	Total
Germany	1	3	74	78
The Netherlands	–	76	–	76
France	61	1	3	65
Slovenia^a^	–	1	41	42
Sweden	–	14	–	14
Austria	–	12	2	14
Denmark	–	6	–	6
Hungary	–	4	–	4
Italy	–	4	–	4
Finland	–	2	–	2
Switzerland	1	1	–	2
Poland	–	–	1	1
Total	63	124	121	308

^a^Two strains mentioned as originating from the former Yugoslavia in the MLST database have been incorporated into the Slovenian strains group

**Table 2:** Clinical manifestations associated with the clinical strains included in this study

Sample	Clinical manifestations	Strains typed in this study	Strains typed by Coipan et al. [15]	Strains extracted from the MLST database	Total
Skin biopsy	EM	43	98	49	190
	MEM	7	–	–	7
	ACA^a^	8	26	7	41
	BL^a^	3	–	5	8
	Morphea	–	–	1	1
	Unspecified	–	–	35	35
CSF	NB^a^	–	–	20	20
Joints	LA^a^	1	–	1	2
Muscle biopsy	Fasciitis^a^	1	–	–	1
Blood	Bacteremia^a^	–	–	1	1
Unknown	–	–	–	2	2
Disseminated manifestations^a^		20	26	34	80
Total		63	124	121	308

*Abbreviations*: EM, erythema migrans; MEM, multiple erythema migrans; ACA, acrodermatitis chronica atrophicans; NB, neuroborreliosis; BL, borrelial lymphocytoma

^a^Manifestations corresponding to a disseminated form of Lyme borreliosis

### Bacterial growth and DNA extraction

The 63 *B. afzelii* strains were obtained from human samples collected between 1997 and 2017. They were grown in modified Barbour-Stoenner-Kelly (BSK-H) medium (Sigma-Aldrich, Saint Quentin Fallavier, France) at 33 °C [23]. DNA extraction from all strains was performed using Chelex-100 resin (Bio-Rad, Marnes-la-Coquette, France), except for four strains (IBS 1, 15, 16, and 17), the DNA of which was extracted using MagNaPure LC DNA Isolation Kit I (Roche Diagnostics, Meylan, France). Genotyping of these *B. afzelii* strains has been previously performed with an in-house real-time polymerase chain reaction (PCR) assay using hybridization probes to target species-specific regions of the *fla* gene [24].

### PCR and sequencing

The primers and conditions of PCR amplification of the eight MLST housekeeping loci (*clpA*, *clpX*, *nifS*, *pepX*, *pyrG*, *recG*, *rplB* and *uvrA*) were used as previously described [13, 25] with one modification: the use of the Phusion High-Fidelity PCR Master Mix (2×) (Thermo Fischer Scientific, Villebon-sur-Yvette, France) with an appropriate denaturing temperature of 98 °C (initial denaturation: 30 s; cycles: 10 s). The success of the amplification was checked by performing agarose gel electrophoresis, followed by cleaning and sequencing the PCR products in both directions (by the firm GATC, Konstanz, Germany). DNA traces were converted into high-quality finished DNA sequences using the SeqTrace software [26]. All sequence data on the 63 strains typed in this study are available on the online MLST database [22].

### Nucleotide sequence analysis

The sequences of the eight housekeeping loci were compared with those in the online MLST database to assign allele and ST numbers. Novel alleles and novel STs were submitted to the curator who allocated them consecutive numbers. The sequence data from the strains obtained from the online MLST database (*n* = 121) and the sequence data from the study by Coipan et al*.* [15] (*n* = 124) were incorporated into the dataset. Multiple sequence alignment of the sequences of individual genes was conducted using the ClustalW algorithm of MEGA 7.0 [27]. The sequences of every individual locus were trimmed to equivalent lengths, as in the *Borrelia* MLST database. Sequences of eight loci were concatenated, giving an in-frame sequence of 4785 bp (not including nucleotide insertions/deletions).

### Genetic diversity

Parameters indicative of population genetic diversity, such as number of alleles and polymorphic sites, nucleotide (π) and haplotype (Hd) diversity, were calculated using DnaSP 5.10 [28].

### Phylogenetic analysis

A maximum likelihood tree based on the concatenated sequences of all the STs under investigation was constructed using the MEGA 7.0 software. The optimal evolutionary nucleotide substitution model used was determined with the corrected Akaike information criterion using the W-IQ-Tree tool available online [29]. The general time reversible model, with gamma distributed rate variation across sites and a proportion of invariable sites, was selected for the phylogenetic analysis (GTR+G+I). The tree was rooted with the European *B. garinii* 20047 strain as the outgroup, extracted from the MLST database. Support for internal nodes was estimated using the nonparametric bootstrap method with 1000 replications.

Allelic profiles of STs were used to generate a global optimal eBurst (goeBURST) diagram using the PHYLOViZ software [30]. This algorithm, based on a set of hierarchical rules relating to the number of single-locus-variants (SLV), double-locus-variants (DLV), or triple-locus-variants (TLV) involved, is appropriate for use with MLST data and can support the identification of relationships between STs in a bacterial population. Both SLV and DLV thresholds were used to identify CCs. Therefore, the STs belonging to a CC share at least 7/8 (SLV) or 6/8 (DLV) identical alleles with at least one other ST in the complex. Major CCs were defined as groups of three or more STs, and minor CCs were defined as two STs. Singletons do not belong to any CC. CCs identified at the SLV threshold were named CC’s.

### Population genetic structure analysis

Pairwise fixation index (F_ST_) values were calculated among the populations of *B. afzelii* defined by the country of origin. Only European countries with a sufficiently large number of strains were considered (Germany, the Netherlands, France, Slovenia, Sweden and Austria). Analyses were conducted using the ARLEQUIN 3.5 program [31] with 1000 permutations to assess the significance of the F_ST_-value. The level of significance decreased from *P* < 0.05 to *P* < 0.0033 using the Bonferroni correction to account for multiple pairwise comparisons. F_ST_-values were interpreted and categorized as follows: 0–0.05 (little genetic differentiation), 0.05–0.15 (moderate genetic differentiation), 0.15–0.25 (great differentiation), and > 0.25 (very great differentiation) [32]. Analysis of molecular variance (AMOVA) was performed using groups of strains per country to analyze the entire population.

### Pathogenicity analysis

In line with previously published classifications, EM was defined as localized infection, and ACA, NB, multiple EM, borrelial lymphocytoma, Lyme arthritis, fasciitis, and bacteremia were all defined as disseminated infection [7, 10]. The single case of morphea reported was not classified because the existence of a causal relationship between this skin manifestation and Lyme disease has not yet been definitely established [33]. In addition, several Slovenian strains extracted from the MLST database were not classified as they were reported as having originated from skin samples without any further explanatory detail. Detailed information about the number of strains belonging to each ST and CC and strains associated with each clinical manifestation is listed in Additional file 2: Database 2. To determine whether some STs or CCs have a propensity to cause disseminated infections, Bayesian techniques [Markov chains and Monte Carlo integrations (MCMC)] were applied with 5000 iterations of the Markov chains after convergence was reached. In keeping with Congdon’s recommendations, a multinomial distribution was assumed for estimating all the cells in contingency tables containing the counts of strains (ST or CC) *vs* clinical features [34]. Minimally informative Dirichlet prior (with parameters 0.5) was assumed on proportion in each cell of tables. This prior was obtained using its Gamma univariate distributions counterpart with rate 1. Lastly, a 2 × 2 table was built using posterior distributions of proportions (the sum of proportions when an aggregated cell was necessary in the table), and the two proportions of interest were compared by monitoring their difference. The probability that one proportion was lower than another (the probability of inferiority) was estimated by counting the number of times that the value of the first proportion adopted in the MCMC process was lower than the corresponding value of the second proportion. When no lower values were returned, the probability was assumed to be < 1/5001 and rounded off to < 0.0002.

## Results

### Intra-species diversity

The MLST analysis of all 308 clinical *B. afzelii* strains included in this study yielded a total of 149 STs (0.48 STs/isolate) (Additional file 1: Database 1). Among these 149 STs, 103 each corresponded to a single strain, whereas 46 STs were each associated with at least two strains. The most prevalent ST was the ST 71 representing 7.5% of the dataset (*n* = 23). Among the 63 strains typed in this study, 10 new alleles and 27 new STs were identified, none of which have been previously described (numbers assigned to the new STs: 695–718; 731–733). The sequence of one of these new alleles (*uvrA* No. 203) includes an insertion consisting of nine nucleotides corresponding to a tandem duplication. The analysis of concatenated sequences resulted in a haplotype diversity of 0.984 and a nucleotide diversity of 0.00202 (Table 3). The highest values obtained corresponded to the locus *pyrG* (0.862 haplotype diversity and 0.00449 nucleotide diversity) and the lowest values were observed in *clpX* (0.188 haplotype diversity and 0.00033 nucleotide diversity).

**Table 3:** Population genetic diversity parameters of *B. afzelii* strains isolated from clinical samples

Locus	No. of nucleotides	No. of alleles	No. of SNPs	No. of indels	Hd ± SD	π ± SD
*clpA*	579	14	11	0	0.762 ± 0.016	0.00199 ± 0.00009
*clpX*	624	7	6	0	0.188 ± 0.030	0.00033 ± 0.00006
*nifS*	564	11	9	0	0.613 ± 0.015	0.00134 ± 0.00007
*pepX*	570	22	16	0	0.849 ± 0.013	0.00297 ± 0.00012
*pyrG*	603	22	14	0	0.862 ± 0.011	0.00449 ± 0.00010
*recG*	651	21	15	0	0.840 ± 0.011	0.00281 ± 0.00008
*rplB*	624	12	9	0	0.401 ± 0.034	0.00088 ± 0.00009
*uvrA*	570	10	8	1^a^	0.613 ± 0.020	0.00135 ± 0.00007
Concatenated sequences	4785	149	88	1	0.984 ± 0.003	0.00202 ± 0.00003

*Abbreviations*: SNP, single nucleotide polymorphism; indels, insertions/deletions; Hd, haplotype diversity; π, nucleotide diversity; SD, standard deviation

^a^Allele 203: insertion of AGATTAAAG between positions 537 and 538 (tandem duplication)

### Phylogenetic analyses

**Fig. 1:**
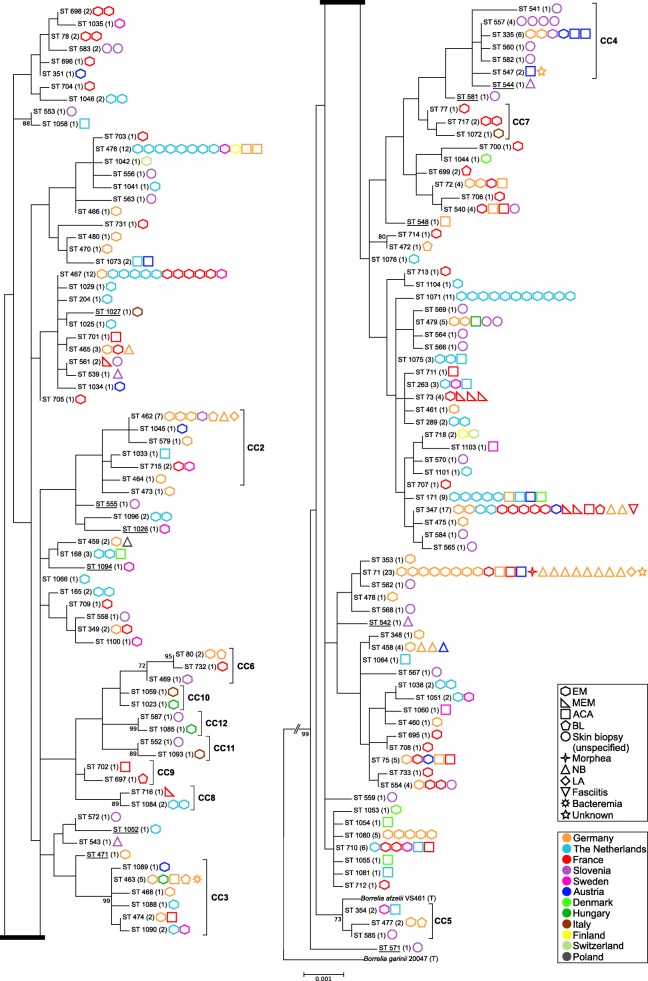
Rooted maximum likelihood tree of *B. afzelii* based on concatenated sequences of eight MLST housekeeping genes. A total of 149 STs corresponding to 308 *B. afzelii* isolates grown *in vitro* from human samples were used in this study, including data previously published by Coipan et al. [15] (*n* = 126) and data from the MLST database (*n* = 121) [13, 22]. The total number of strains associated with each ST is indicated in brackets next to the ST. Type strains (T) of *B. afzelii* (strain VS461) and *B. garinii* (strain 20047) were also included in the dataset. The bootstrap values obtained on highly supported nodes after 1000 repetitions (with ≥ 70% support) are given below the branches. The grouping of STs into CCs defined by goeBURST analysis at the DLV threshold is indicated by brackets in the case of CC1 to CC12. STs corresponding to singletons are underscored. All other STs belong to the CC0 distributed throughout the tree. The type of infection associated with each strain is indicated by a geometrical shape next to the STs, whereas the geographical origin is indicated by a color (see the legend). Black rectangles give a section of the tree for the sake of better legibility

Phylogenetic analyses were based on concatenated sequences of type strains belonging to each *Borreliella* species extracted from the MLST database. The analyses confirmed that the 63 strains typed using MLST in this study were definitely *B. afzelii* strains (Additional file 3: Figure S1). The rooted phylogenetic tree based on concatenated sequences of all the included clinical *B. afzelii* strains showed short evolutionary distances between strains, and few bootstrap values were > 70% (Fig. 1). No major clade was detected. The clinical manifestations and countries of origin related to the strains were distributed throughout the phylogenetic tree.

**Fig. 2:**
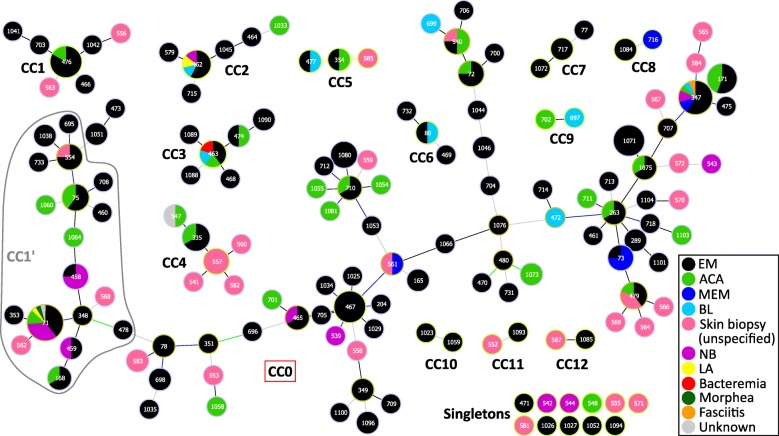
Overview of the relationships between European *B. afzelii* STs detected in human samples using goeBURST. CCs were identified at the DLV threshold. The 149 STs clustered into eight major complexes (consisting of three or more STs), five minor complexes (consisting of two STs), and 11 singletons with no connection with any other STs. The circled fraction of CC0 corresponds to CC1’ when CCs were defined at the SLV threshold. Colored lines between STs indicate in descending order of certainty: black lines inferred without tiebreak rules, blue lines inferred using tiebreak rule 1 (number of SLVs), green lines using tiebreak rule 2 (number of DLV), and yellow lines using tiebreak rules 4 or 5 (frequency found on the data set and ST number, respectively). STs connected by gray lines are DLVs. The inferred founders of CCs are outlined in light green and subgroup founders in dark green. Circle size corresponds to MLST sample size. Circle color fractions refer to clinical manifestations associated with STs (see the legend)

A goeBURST analysis provided a population snapshot of clinical *B. afzelii* strains, as shown in Fig. 2. At the DLV threshold, eight major CCs (consisting of three or more STs), five minor CCs (consisting of two STs), and 11 singletons were identified. The largest CC, named CC0, comprised 94 STs distributed throughout the phylogenetic tree, based on the concatenated sequences of the eight MLST genes (Fig. 1). Three major CCs (CC3, CC5, and CC6) and three minor CCs (CC8, CC11, and CC12) clearly formed highly supported terminal clusters on this tree (bootstrap values ≥ 70%). Except for CC0, all the other CCs also formed clusters on the tree but did not reach statistically significant values (bootstrap values < 70%). In contrast, the use of the SLV threshold yielded 13 major CC’s, seven of which belonged to CC0 at the DLV threshold. Seven minor CC’s and 40 singletons were also identified using this method.

### Spatial distribution of STs detected in European Lyme borreliosis patients

**Fig. 3:**
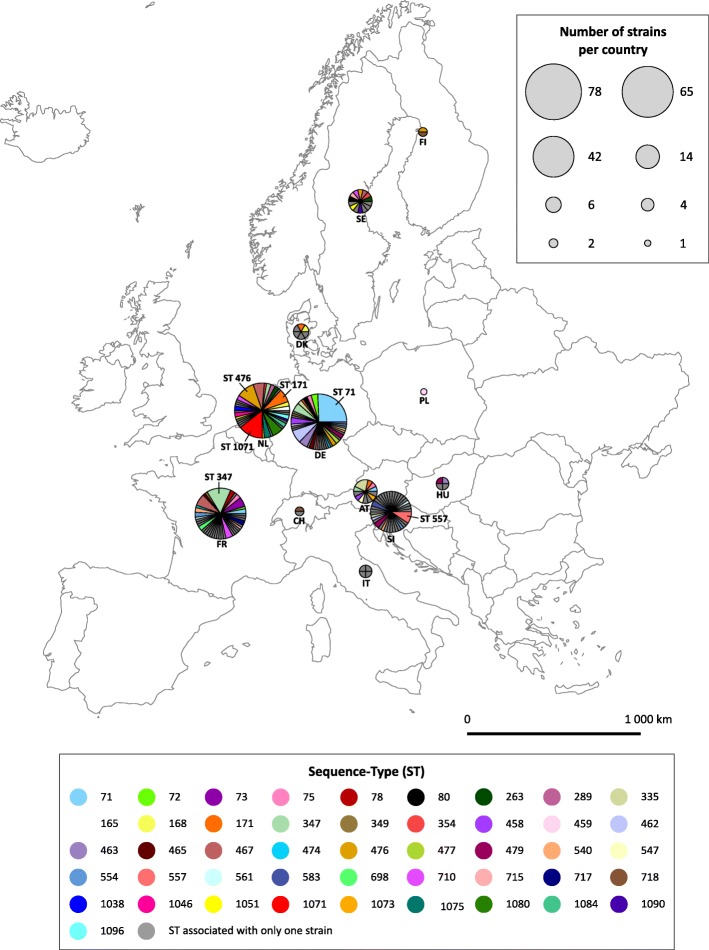
Spatial distribution of STs involving the European *B. afzelii* strains included in this study. Pie charts showing the distribution of the STs involving the 308 *B. afzelii* strains included in this study in Germany (DE), the Netherlands (NL), France (FR), Slovenia (SI), Austria (AT), Sweden (SE), Denmark (DK), Hungary (HU), Italy (IT), Switzerland (CH) and Poland (PL). The size of each sector is proportional to the total number of strains corresponding to each country (see box). Main STs in the four countries showing the largest numbers of strains (DE, NL, FR and SI) are indicated on the map

In total, 27 of the 46 STs that occurred in more than one patient were isolated in at least two different European countries (Additional file 1: Database 1). Patients from Slovenia had a greater ST diversity (0.88 STs/strain) than those from Germany, The Netherlands, and France (0.47, 0.62, and 0.46 STs/strain, respectively). The main STs differed between the four countries most frequently involved, namely Germany (ST 71), the Netherlands (STs 1,071, 476 and 171), France (ST 347) and Slovenia (ST 557) (Fig. 3). The pairwise comparisons between population samples suggested moderate genetic differentiation between strains from the Netherlands and Germany (F_ST_ = 0.08975, *P* < 0.0001), the Netherlands and Slovenia (F_ST_ = 0.05631, *P* < 0.0001), and the Netherlands and Austria (F_ST_ = 0.07706, *P* < 0.0001) (Table 4). The other F_ST_ values were < 0.05 showing the existence of low genetic differentiation (Table 4). The overall F_ST_ value was 0.04405, based on an AMOVA performed on all 308 strains analyzed per country (Table 5). Less than 5% (4.4%) of the total genetic variance was attributable to genetic differentiation among populations from the various European countries.

**Table 4:** Matrix of pairwise F_ST_ values of the STs in various European countries. Analyses were not conducted on countries associated with too few clinical strains (i.e. Denmark, Hungary, Italy, Finland, Switzerland and Poland)

	Germany	The Netherlands	France	Slovenia	Sweden
	F_ST_ (*P*-value)	F_ST_ (*P*-value)	F_ST_ (*P*-value)	F_ST_ (*P*-value)	F_ST_ (*P*-value)
The Netherlands	**0.08975** **(< 0.00001)**	–	–	–	–
France	**0.03957** **(< 0.00001)**	0.02663 (0.00684)	–	–	–
Slovenia	**0.04417** **(< 0.00001)**	**0.05631** **(< 0.00001)**	0.01369 (0.06348)	–	–
Sweden	0.02722 (0.06934)	0.02356 (0.11133)	-0.00526 (0.56836)	0.02075 (0.10840)	–
Austria	0.02527 (0.09766)	**0.07706** **(< 0.00001)**	0.01494 (0.18555)	-0.02581 (0.95801)	0.02188 (0.17969)

Values in bold type were significant at a significance threshold of α = 0.0033

**Table 5:** Results of the analysis of molecular variance (AMOVA) using strains arranged per country

	df	Percentage variation	Mean F-statistic over loci	*P*-value
Among countries	11	4.4	F_ST_ = 0.04405	< 0.00001
Within countries	296	95.6	–	–

*Abbreviation*: df, degrees of freedom

### STs associated with clinical manifestations of human Lyme borreliosis

Among the 308 strains included in this study, a total of 270 strains were classified as associated with localized or disseminated infection (Table 2, Additional file 2: Database 2). The 38 remaining strains were not classifiable since they originated from a morphea (*n* = 1), from unspecified cutaneous samples (*n* = 35) and from samples of unknown nature (*n* = 2). Among the 270 strains considered, 190 (70.4%, corresponding to 41 STs) were collected from patients with a localized infection (EM), whereas 80 (29.6%, corresponding to 24 STs) were associated with manifestations suggesting disseminated infection. Most of the *B. afzelii* strains isolated from patients with disseminated infections were involved in skin damage, including ACA, borrelial lymphocytoma, and multiple EM (*n* = 56, 70%). Only 20 strains were isolated from cerebrospinal fluid in the context of NB.

Among the 42 STs detected in at least two patients with localized or disseminated manifestations, 18 STs were present only in patients with localized infection (EM) (Table 6, Additional file 2: Database 2). In particular, ST 467 and ST 1071 were associated with 12 and 11 strains obtained from patients with EM, respectively, and no strain was associated with disseminated infection. ST 467 and ST 1071 were significantly related to localized infection compared with other STs that were associated with at least two strains (EM: 100 *vs* 66.67% [95% CI: 59.23–73.23%] and 100 *vs* 66.90% [95% CI: 59.59–73.32%], respectively; probability of inferiority < 0.0002). Conversely, ST 1073 was the only ST found solely in patients with disseminated infection (two cases of ACA), but the number of strains associated with it (*n* = 2) was too small to be able to draw any definite conclusions regarding invasiveness. We also noted that ST 71, which was associated with the largest number of strains (*n* = 23), was involved in 12 cases of disseminated infection and nine cases of localized infection (two strains were not classified). The other STs associated with least two strains were less frequently involved in disseminated manifestations (*n* = 46) than in EM symptoms (*n* = 122). This difference was statistically significant (disseminated infection: 58.26% [95% CI: 37.61–76.44%] *vs* 27.87% [95% CI: 21.81–35.73%]; probability of inferiority = 0.003). More specifically, ST 71 was significantly involved in NB (*n* = 8) *vs* EM compared with the other STs associated with at least two strains (NB: 48.27% [95% CI: 26.63–69.73%] *vs* 5.88% [95% CI: 2.84–10.55%]; probability of inferiority < 0.0002).

**Table 6:** Distribution of STs between patients with localized and disseminated infection. Only STs identified in two or more patients were included

ST	Total no. of strains	Localized infection	Disseminated infection
71	21	9	12
72	4	3	1
73	4	1	3
75	5	3	2
78	2	2	0
80	2	1	1
165	2	2	0
168	3	2	1
171	9	5	4
263	3	2	1
289	2	2	0
335	6	4	2
347	17	10	7
349	2	2	0
354	2	1	1
458	4	1	3
459	2	1	1
462	7	4	3
463	5	2	3
465	3	2	1
467	12	12	0
474	2	1	1
476	12	10	2
477	2	1	1
479	3	2	1
540	3	1	2
554	3	3	0
698	2	2	0
710	6	4	2
715	2	2	0
717	2	2	0
718	2	2	0
1038	2	2	0
1046	2	2	0
1051	2	2	0
1071	11	11	0
1073	2	0	2
1075	3	2	1
1080	5	5	0
1084	2	2	0
1090	2	2	0
1096	2	2	0
Total	189	131	58

No association was reported between clinical manifestations and the CCs identified at the DLV threshold. STs 71 (associated with disseminated infection), 467, and 1071 (associated with EM) all belong to the CC0 comprising 94 STs, which makes it impossible to draw any significant conclusions (Additional file 2: Database 2). However, when the CCs were defined at the SLV threshold, CC1’, including ST 71, was found to be significantly involved in NB compared with other CC’s (NB: 30.31% [95% CI: 18.62–47.27%] *vs* 3.50% [95% CI: 1.26–9.49%]; probability of inferiority < 0.0002) (Table 7).

**Table 7:** Distribution of clonal complexes between patients with erythema migrans and neuroborreliosis. Clonal complexes were defined by performing goeBURST analysis at the SLV level. The CCs defined at the SLV threshold were named CC’s. Thirteen major CC’s and seven minor CC’s were identified. The 40 singletons identified were not included

CC’ (SLV)	EM	NB
CC0’ ^a^	42	2
CC1’ ^a^	27	12
CC2’ ^a^	21	2
CC3’ ^a^	11	0
CC4’ ^a^	4	0
CC5’ ^a^	6	0
CC6’ ^a^	7	0
CC7’ ^a^	6	0
CC8’ ^a^	13	0
CC9’ ^a^	7	1
CC10’ ^a^	2	0
CC11’ ^a^	2	0
CC12’ ^a^	3	0
CC13’	0	0
CC14’	0	0
CC15’	3	0
CC16’	1	0
CC17’	3	0
CC18’	1	0
CC19’	2	0

^a^Thirteen major CC’s

## Discussion

This study was focused on *B. afzelii*, the main species involved in Lyme borreliosis in humans in Europe [35], with the aim to investigate the European strains of *B. afzelii* obtained from human samples to determine their diversity, spatial distribution, and pathogenicity using their MLST profiles. A total of 308 *B. afzelii* strains were included in the dataset. Sixty-three of these strains, mostly originating from France, were characterized using MLST, and sequence data of additional 245 strains originating from several European countries were accessed from the literature [15, 22].

The 308 *B. afzelii* strains obtained from European human patients were categorized into 149 STs, 27 of which were described for the first time in this study. These data reported 0.48 STs/strain (0.88 STs/strain in Slovenia) highlighting the high level of genetic heterogeneity among the *B. afzelii* strains responsible for Lyme borreliosis in Europe. This value is fairly similar to that reported by Coipan et al*.* [15] on 135 clinical *B. afzelii* strains in Europe (0.50 STs/strain); data on 124 of these strains were included in this study’s dataset. In addition, the reported high levels of haplotype diversity and low levels of nucleotide diversity in our study were also supported by the results reported by Coipan (0.984 *vs* 0.976 and 0.00202 *vs* 0.00196, respectively). Although a larger number of strains was included in our study, this did not affect the validity of the genetic heterogeneity parameters previously described. In addition, Coipan et al*.* [15] conducted a rarefaction analysis, which did not show the evidence of any significant difference in *B. afzelii* diversity between humans and ticks, measured in terms of the number of STs. The authors observed a high level of haplotype diversity and a low level of nucleotide diversity among the *B. afzelii* strains found in ticks (0.978 and 0.00292, respectively). These findings suggest that the European population of *B. afzelii* has undergone a bottleneck effect before expanding, resulting in the frequent occurrence of single mutations [36].

In the phylogenetic tree obtained in this study, short evolutionary distances were observed between STs, with few bootstrap values ≥ 70% and no major clade. These findings are consistent with the presence of a large major CC (CC0) distributed throughout the tree, comprising 94 of the 149 STs, demonstrating that the strains included are closely related. In addition, defining CCs *via* goeBURST based on allele identity rather than sequence diversity tends to limit the impact of horizontal gene transfer events on the identification of clusters of closely related organisms. Genetic events of this type may have occurred occasionally in *Borreliella* spp., even in the housekeeping genes targeted by the MLST [14, 15, 37]. However, although the CCs defined at the DLV threshold were not all supported statistically by the phylogenetic tree, none of them, except CC0, split into several branches. The clinical manifestations and countries of origin associated with all the strains were dispersed throughout the phylogenetic tree.

The countries representing the largest number of strains in this study were Germany, the Netherlands, France and Slovenia, accounting for 85% of all the strains under investigation. More than half of the STs encountered at least twice were identified in several European countries (27/46), suggesting that little population differentiation has occurred among the *B. afzelii* strains infecting humans in Europe. However, in a study on the MLST profiles of 72 *B. afzelii* strains identified in ticks in four European countries (England, France, Germany and Latvia), Vollmer et al*.* [38] reported a pronounced spatial differentiation as only two of the 40 STs were identified in more than one country. Likewise, the population genetic structure assessed using pairwise F_ST_-values was weaker in the present study than that previously described by Vollmer et al*.* [38] in strains isolated from ticks. Here we observed only low to moderate genetic differentiation with a maximal pairwise F_ST_-value of 0.090 (Germany/The Netherlands), whereas Vollmer et al. previously reported values ranging from 0.080 (Germany/Latvia) to 0.364 (England/Latvia), without any tendency to increase with geographical distance. The only pair of countries that was investigated in both studies was Germany/France. Our study reported a pairwise F_ST_-value of 0.040 compared with 0.118 in that by Vollmer et al. [38]. The lower value in our study might be explained by sites that were in proximity to each other on both sides of the border between Germany and France. However, this explanation is unlikely to hold true as most of the German *B. afzelii* strains originated more centrally from Munich. In addition, the AMOVA resulted in an overall F_ST_-value of 0.044, which is also much lower than the value of 0.222 published by previous authors [38]. This difference suggests that *B. afzelii* strains isolated from humans in Europe are genetically closer than strains occurring in ticks. Previously reported data showed that some genotypes of *B. afzelii* and *B. burgdorferi* have a greater tendency to cause Lyme borreliosis in humans [15, 18]. On similar lines, it has been proposed that the diversity of *B. burgdorferi* strains is significantly greater in ticks than in the skin of patients with EM, suggesting that human skin acts as a filter, thus allowing entry to only a fraction of the total population [39]. However, care must be exercised when directly comparing the results reported in this study with those of Vollmer et al. [38], which included much fewer (*n* = 72) *B. afzelii* strains and involved different European countries, in particular England, which was not represented in the current study [38]. Although the results of the current study indicate only low to moderate genetic differentiation between clinical *B. afzelii* strains from Europe, this genetic differentiation was still significant. This could be because *B. afzelii* has a host association with rodents, which perform little migration, whereas both the bird-related species *B. garinii* and *B. valaisiana* are associated with spatial mixing of STs between European countries [38, 40, 41].

One of the objectives of the present study was to determine whether there are lineages of *B. afzelii* with differential pathogenicity to humans. The 308 strains included were mostly isolated from skin biopsies, in agreement with previous data showing that *B. afzelii* is strongly associated with ACA and cutaneous manifestations in general [10, 20, 42]. This study did identify some MLST profiles of *B. afzelii* with specific dissemination properties in humans. We decided to examine only the 42 STs detected in at least two patients with localized or disseminated infection for the sake of comparison, as per the methods of previous authors [14, 20]. Two STs (ST 467 and ST 1071) were significantly associated with EM but not involved in manifestations of any other type, suggesting a relatively weak ability to disseminate. Conversely, ST 71 was significantly associated with disseminated manifestations, and most of these strains were involved in NB. All three of these STs (ST 467, ST 1071, and ST 71) were associated with more than 10 strains. It is worth noting that to date, ST 71 has never been found in ticks positive for *B. afzelii* (*n* = 319 ticks) [15, 22]. ST 71 also belongs to a CC called CC1’, defined at the SLV threshold, which was significantly associated with NB compared with other CC’s. Other associations between *B. afzelii* STs and disseminated forms of Lyme borreliosis in Europe were previously described by Coipan et al*.* [15] using a smaller dataset of 135 clinical strains. They established a significant association between ST 335, ST 1054, ST 1073 and ACA (cases of ACA *vs* EM: 2/1, 2/0, and 2/0, respectively). These conclusions were not reinforced by our study, although our dataset was more than twice the size of that used in the previous study. We did not observe any supplementary strains corresponding to these STs, apart from the three strains belonging to ST 335 and associated with EM. However, we noticed that some *B. afzelii* strains were more frequently associated with disseminated symptoms based on their MLST profiles. The MLST procedure is based on the sequencing of eight housekeeping genes, which, by definition, are not directly involved in pathogenicity. Thus, it seems likely that differences in the dissemination properties of some lineages of *Borreliella* spp. revealed using MLST are due to a strong linkage disequilibrium between targeted chromosomal genes and the virulence factors encoded by chromosomal or plasmid genes. The imperfect associations that were found between STs and pathogenicity may be attributable to recombination events, which might decrease this linkage disequilibrium [37]. The immunity of hosts and possible co-infections with other pathogens may also be key factors involved in the dissemination of *Borreliella* spp. in humans [43, 44].

Some genotypes of *B. afzelii* may have been selected during the *in vitro* growth of strains, which would affect the representativeness of the dataset, thus introducing a possible bias. This bias could also hide some mixed-strain infections. More studies wherein MLST is performed on DNA extracts from clinical samples, instead of strain cultures, would help to establish whether this selection of genotypes actually exists and to what extent it could impact our results.

## Conclusions

The comparisons of MLST profiles showed a low genetic differentiation among *B. afzelii* strains isolated from patients with Lyme borreliosis in Europe. Clinical data analysis suggests the existence of some lineages with differential dissemination properties in humans. Comparative genomic studies between MLST lineages with different degrees of pathogenicity would help to complete our understanding of the bacterial factors contributing to the invasiveness of *B. afzelii* strains.

## Additional files

Additional file 1: **Database 1.** Detailed information relating to strains included in this study. All strains are listed, including those typed for this study, those extracted from the online database, and those sequenced by Coipan et al. [15]. Information is provided on the geographical origin, the clinical symptom, and sequence data relating to each strain. A list featuring the countries where each ST was identified is also available. (XLSX 103 kb)

Additional file 2 **Database 2.** Clinical manifestations of Lyme borreliosis associated with strains grouped per ST and CC. Analyses were conducted on CCs using both DLV and SLV thresholds. (XLSX 78 kb)

Additional file 3: **Figure S1.** Maximum likelihood phylogenetic tree based on concatenated sequences of strains typed for this study and typed strains belonging to each *Borreliella* species. The 63 strains typed for this study were included in the dataset. They were previously identified as belonging to *B. afzelii*. Typed strains (T) of each *Borreliella* species were also included in the analysis with concatenated sequences extracted from the online MLST database. The bootstrap values were obtained after 1000 repetitions. (PDF 19 kb)

## Abbreviations

MLST: multilocus sequence typing
ST: sequence types
EM: erythema migrans
NB: Lyme neuroborreliosis
ACA: acrodermatitis chronica atrophicans
CC: clonal complex
