# Supplementary figures and images for: Multilocus sequence typing of clinical Borreliella afzelii strains: population structure and differential ability to disseminate in humans
Source: Parasit Vectors. 2018 Jun 28;11:374. doi: 10.1186/s13071-018-2938-x (PMC6027761; doi:10.1186/s13071-018-2938-x)

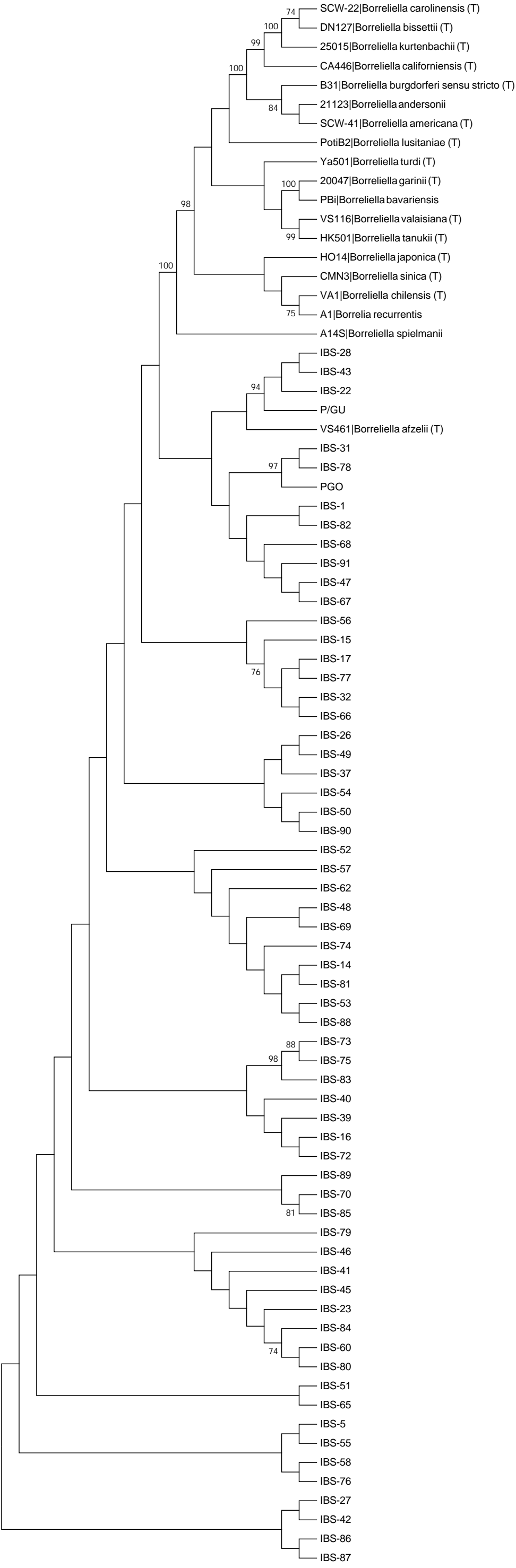

Supplement: Supplementary file 3 — Figure S1. Maximum likelihood phylogenetic tree based on concatenated sequences of strains typed for this study and typed strains belonging to each Borreliella species. The 63 strains typed for this study were included in the dataset. They were previously identified as belonging to B. afzelii. Typed strains (T) of each Borreliella species were also included in the analysis with concatenated sequences extracted from the online MLST database. The bootstrap values were obtained after 1000 repetitions. (PDF 19 kb) [file 13071_2018_2938_MOESM3_ESM.pdf]
